# JEWELFISH: 24-month results from an open-label study in non-treatment-naïve patients with SMA receiving treatment with risdiplam

**DOI:** 10.1007/s00415-024-12318-z

**Published:** 2024-05-11

**Authors:** Claudia A. Chiriboga, Claudio Bruno, Tina Duong, Dirk Fischer, Eugenio Mercuri, Janbernd Kirschner, Anna Kostera-Pruszczyk, Birgit Jaber, Ksenija Gorni, Heidemarie Kletzl, Imogen Carruthers, Carmen Martin, Renata S. Scalco, Paulo Fontoura, Francesco Muntoni, the JEWELFISH Study Group

**Affiliations:** 1 Department of Neurology, Columbia University Irving Medical Center, New York, NY USA; 2 Centre of Translational and Experimental Myology, IRCCS Istituto Giannina Gaslini, Genoa, Italy; 3 Department of Neuroscience, Rehabilitation, Ophthalmology, Genetics, Maternal and Child Health-DINOGMI, University of Genoa, Genoa, Italy; 4 Department of Neurology, Stanford University, Palo Alto, CA USA; 5 Division of Neuropediatrics, University Children’s Hospital Basel, University of Basel, Basel, Switzerland; 6 Pediatric Neurology Institute, Catholic University and Nemo Pediatrico, Fondazione Policlinico Gemelli IRCCS, Rome, Italy; 7 Department of Neuropediatrics and Muscle Disorders, Faculty of Medicine, Medical Center-University of Freiburg, Freiburg, Germany; 8 Department of Neurology, Medical University of Warsaw, ERN EURO-NMD, Warsaw, Poland; 9 Pharma Development, Safety, F. Hoffmann-La Roche Ltd, Basel, Switzerland; 10 PDMA Neuroscience and Rare Disease, F. Hoffmann-La Roche Ltd, Basel, Switzerland; 11 Roche Pharmaceutical Research and Early Development, Roche Innovation Center Basel, Basel, Switzerland; 12 Roche Products Ltd, Welwyn Garden City, UK; 13 Product Development Neuroscience, F. Hoffmann-La Roche Ltd, Basel, Switzerland; 14 The Dubowitz Neuromuscular Centre, NIHR Great Ormond Street Hospital Biomedical Research Centre, Great Ormond Street Institute of Child Health University College London, and Great Ormond Street Hospital Trust, London, UK

**Keywords:** Spinal muscular atrophy, Risdiplam, SMA, Safety, Motor function

## Abstract

**Abstract:**

Risdiplam is a once-daily oral, survival of motor neuron 2 (*SMN2*) splicing modifier approved for the treatment of spinal muscular atrophy (SMA). JEWELFISH (NCT03032172) investigated the safety, tolerability, pharmacokinetics (PK), and PK/pharmacodynamic (PD) relationship of risdiplam in non-treatment-naïve patients with SMA. JEWELFISH enrolled adult and pediatric patients (*N* = 174) with confirmed diagnosis of 5q-autosomal recessive SMA who had previously received treatment with nusinersen (*n* = 76), onasemnogene abeparvovec (*n* = 14), olesoxime (*n* = 71), or were enrolled in the MOONFISH study (NCT02240355) of the splicing modifier RG7800 (*n* = 13). JEWELFISH was an open-label study with all participants scheduled to receive risdiplam. The most common adverse event (AE) was pyrexia (42 patients, 24%) and the most common serious AE (SAE) was pneumonia (5 patients, 3%). The rate of AEs and SAEs decreased by > 50% from the first to the second year of treatment, and there were no treatment-related AEs that led to withdrawal from treatment. An increase in SMN protein in blood was observed following risdiplam treatment and sustained over 24 months of treatment irrespective of previous treatment. Exploratory efficacy assessments of motor function showed an overall stabilization in mean total scores as assessed by the 32-item Motor Function Measure, Hammersmith Functional Motor Scale—Expanded, and Revised Upper Limb Module. The safety profile of risdiplam in JEWELFISH was consistent with previous clinical trials of risdiplam in treatment-naïve patients. Exploratory efficacy outcomes are reported but it should be noted that the main aim of JEWELFISH was to assess safety and PK/PD, and the study was not designed for efficacy analysis.

**Trial registration:**

The study was registered (NCT03032172) on ClinicalTrials.gov on January 24, 2017; First patient enrolled: March 3, 2017.

**Supplementary Information:**

The online version contains supplementary material available at 10.1007/s00415-024-12318-z.

## Introduction

Spinal muscular atrophy (SMA) is a genetic disorder that affects motor neurons, resulting in muscle weakness [1]. It is caused by mutations in the survival of motor neuron 1 (*SMN1*) gene that lead to low levels of functional SMN protein [2].

Three disease-modifying therapies (DMTs) are currently approved for the treatment of SMA: nusinersen (SPINRAZA^®^), an intrathecally administered antisense oligonucleotide that alters the splicing of *SMN2* pre-mRNA [3, 4]; onasemnogene abeparvovec (ZOLGENSMA^®^), an intravenously administered gene therapy designed to deliver a functional copy of the *SMN1* gene into motor neurons [5, 6]; and risdiplam (EVRYSDI^®^), an orally administered small molecule designed to selectively modify splicing of *SMN2* pre-mRNA and promote inclusion of exon 7 thus increasing levels of functional SMN protein [7, 8].

With the availability of three treatment options, treatments may be administered sequentially or in combination [9]. Patients may wish to switch treatments or explore a combination of therapies if they do not perceive a benefit from their treatment or due to patient preferences regarding routes of administration [10–12].

JEWELFISH was the first clinical trial to investigate sequential treatment of SMA with DMTs. Furthermore, there are limited data from real-world clinical settings that evaluated the safety and efficacy of combination or sequential treatment [13–16].

The JEWELFISH study was designed to assess the safety, tolerability, pharmacokinetics (PK), and PK/pharmacodynamic (PD) relationship of risdiplam in non-treatment-naïve adult and pediatric patients with SMA. The interim results after 12 months of treatment have previously been reported [17]. Here we report the primary analysis after 24 months of risdiplam treatment.

## Methods

### Standard protocol approvals, registrations, and participant consent

The study was initiated after institutional review board approvals of the participating study centers, in accordance with the principles of the Declaration of Helsinki and in full conformance with Good Clinical Practice guidelines. The names of these independent ethics committees/institutional review boards have been previously published [17]. An independent Data Monitoring Committee monitored the risdiplam studies on an ongoing basis. The study was registered with ClinicalTrials.gov (NCT03032172). Participants or their legally authorized representatives completed a statement of informed consent. When possible, assent was collected from participants < 18 years of age. The study protocol and statistical analysis plan are available as Online Resource 1 and Online Resource 2, respectively.

### Study design and participants

JEWELFISH is a multicenter, open-label study of daily risdiplam that includes patients with SMA previously enrolled in the MOONFISH study (NCT02240355) [18], or those treated with nusinersen, onasemnogene abeparvovec, or olesoxime [19]. Patients previously treated with nusinersen were included if they had received ≥ 4 doses of nusinersen, provided that the last dose was received ≥ 90 days prior to screening. Patients previously treated with onasemnogene abeparvovec were included provided that the time of treatment was ≥ 12 months prior to screening. Patients previously treated with olesoxime were included provided that the last dose was received ≤ 18 months and ≥ 90 days prior to screening. Patients aged 6 months to 60 years of age at screening were eligible if they had a confirmed diagnosis of 5q-SMA, including genetic confirmation of a biallelic mutation (homozygous deletion or heterozygosity predictive of loss of function of the *SMN1* gene) and clinical history, signs, or symptoms attributable to SMA. Patients were excluded if they met any of the following criteria: participation in any investigational drug or device study with the exception of studies of olesoxime, nusinersen, or onasemnogene abeparvovec ≥ 90 days prior to screening; any history of gene or cell therapy with the exception of onasemnogene abeparvovec; initiation of an oral salbutamol or oral β2-adrenergic agonist within 6 weeks prior to enrollment; any prior use of FMO1 or FMO3 inhibitor or inducer, or OCT-2 and MATE substrates within 2 weeks prior to risdiplam treatment; any prior use of any agents anticipated to increase or decrease muscle strength 90 days prior to enrollment or medications known or suspected of causing retinal toxicity within 1 year prior to enrollment. Patients were excluded if they had a recent history (< 1 year) of ophthalmologic diseases that would interfere with study assessments. Patients aged < 2 years were excluded if they had been hospitalized for a pulmonary event within 2 months prior to screening and pulmonary function had not fully recovered. There were no exclusion criteria based on SMA type, *SMN2* copy number, baseline motor function, comorbidities, or respiratory/feeding support. Patients > 2 years of age were considered ambulant if they had the ability to walk unassisted for ≥ 10 m. Full inclusion and exclusion criteria for JEWELFISH have been published [17]. Based upon practical considerations the study size was set at 180 patients [17]. Assuming the underlying adverse event (AE) rate is 1.4%, a study of 180 patients exposed to risdiplam would have a 92% chance of detecting an AE in ≥ 1 patient. Patients received risdiplam orally once daily at the approved dosing regimen based on body weight and age. For patients aged 2–60 years, the dose is 5 mg for patients with a body weight of ≥ 20 kg, and 0.25 mg/kg for a body weight < 20 kg. For patients aged 6 months to < 2 years, the dose is 0.2 mg/kg [17].

Patients were enrolled across 24 different centers in Belgium, France, Germany, Italy, the Netherlands, Poland, Switzerland, the UK, and the USA. After 24 months of treatment, participants were invited to participate in the open-label extension phase to continue treatment for up to an additional 3 years.

### Study assessments and outcomes

The primary objectives were to evaluate the safety and tolerability of risdiplam and to investigate the PK of risdiplam and metabolites, as appropriate. The secondary objective was to investigate the PK/PD relationship of risdiplam. Investigation of the PD included the analyses of *SMN2* mRNA splice forms and SMN protein.

Key exploratory objectives included evaluations of the efficacy of treatment with risdiplam in terms of motor and respiratory function. Functional assessments were dependent on the age of the patients. Patients aged 2–60 years were assessed using the 32-item Motor Function Measure (MFM32), the Hammersmith Functional Motor Scale—Expanded (HFMSE), and the Revised Upper Limb Module (RULM). Following the start of the study, the 6-min walk test (6MWT) was added as an exploratory endpoint for ambulant patients 6–60 years of age and therefore not all patients have baseline measurements. Patients aged ≤ 2 years were assessed using the Bayley Scales of Infant and Toddler Development, third edition (BSID-III), and achievement of motor milestones was assessed through the Hammersmith Infant Neurological Examination, Module 2 (HINE-2).

Respiratory function was assessed in patients aged 6–60 years by spirometry tests and patients aged 2–60 years performed the sniff nasal inspiratory pressure test. Patient-reported independence in activities of daily living among patients aged 12–60 years and caregiver-reported independence for patients aged 2–60 years were assessed through the SMA Independence Scale–Upper Limb Module (SMAIS–ULM). This assessment was implemented after the study started and, therefore, not all patients have a baseline assessment.

Safety data were reported for all patients and by previous treatment. Additional methods and exploratory efficacy data are reported in Online Resource 3.

### Post hoc safety comparison

A post hoc safety analysis was conducted to compare the rates of AEs and serious AEs (SAEs) over time in patients who received risdiplam in the JEWELFISH study with patients who were treatment-naïve prior to receiving risdiplam in the SUNFISH (NCT02908685) [20] and FIREFISH (NCT02913482) [21] studies.

### Statistical analysis

All patients who received ≥ 1 dose of risdiplam were included in the safety analysis population. The intent-to-treat (ITT) population was defined as all enrolled patients, regardless of whether they received risdiplam or not. The ITT population was used for all exploratory efficacy analyses.

## Results

### Patients

In this analysis, patients had received risdiplam for ≥ 24 months by the clinical cut-off date (CCOD) of January 31, 2022. Between March 2017 and January 2020, a total of 182 patients were screened for eligibility with 174 participants meeting the recruitment criteria. Of the 174 patients enrolled in the study, one patient previously treated with olesoxime withdrew from the study at baseline prior to receiving risdiplam due to poor venous access as assessed by their physician. A total of 173 patients received risdiplam.

**Fig. 1:**
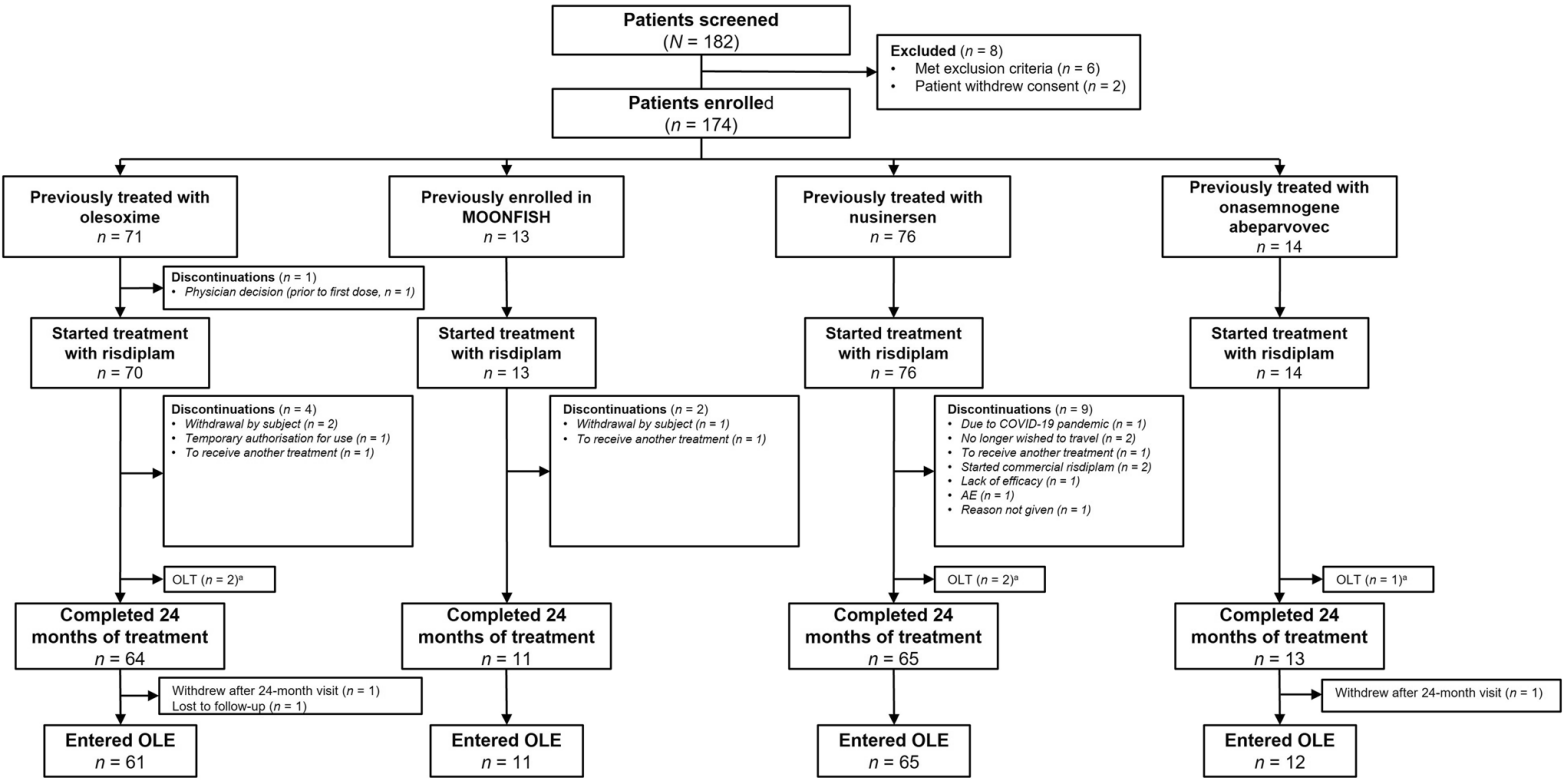
Participant flow diagram. *AE* adverse event, *CCOD* clinical cut-off date, *COVID-19* coronavirus disease 2019, *OLE* open-label extension, *OLT* open-label treatment. ^a^Two nusinersen, one onasemnogene abeparvovec, and two olesoxime patients did not attend the week 104 visit and, therefore, as of the CCOD, are still represented as being in the OLT period

Patients were grouped according to their previous treatment types: 71 patients were previously treated with olesoxime in the OLEOS study (NCT02628743); 13 patients had previously been enrolled in the MOONFISH study, 10 of whom received RG7800 (RO6885237) and three who received placebo; 76 patients were previously treated with nusinersen, three of whom had also previously received olesoxime; 14 patients were previously treated with onasemnogene abeparvovec. One patient in the onasemnogene abeparvovec group had also received subsequent treatment with nusinersen. A total of 153 patients (87.9%) completed 24 months of treatment. Fifteen patients withdrew prior to the completion of 24 months of treatment, five patients did not attend the week 104 visit as of the CCOD and therefore are still represented as being in the open-label treatment period and one patient never received treatment (Fig. 1).

### Baseline characteristics

The patient population was broad and heterogeneous, covering a wide range of ages and baseline motor functions, and included patients with a high degree of motor impairment and other SMA comorbidities.

The median age of the JEWELFISH population at enrollment was 14 years (range 1–60); the median age at enrollment for patients who previously received nusinersen or onasemnogene abeparvovec was 12.0 years (range 1–60 and 2.0 years (range 1–5), respectively. The majority of patients had three *SMN2* copies (78%). The median duration from symptom onset to first risdiplam administration was 134.5 months for patients who previously received nusinersen and 29 months for patients who previously received onasemnogene abeparvovec. The median time to first risdiplam administration from the last nusinersen dose was 4.6 months (range 3.5–19.3) and from treatment with onasemnogene abeparvovec was 16.8 months (range 13.3–21.4). In the subgroup of patients aged 2–60 years, 83% had scoliosis and 48% had received scoliosis surgery. Additionally, 63% had a baseline total score of <10 on the HFMSE. Detailed baseline characteristics can be found in Table 1. Fifteen patients with Type 3 SMA were ambulatory and had a median age at enrollment of 15 years (range 5–46).

**Table 1:** Key patient demographics at baseline

	Previous treatment				
	Olesoxime (*n* = 71)	MOONFISH study (RG7800) (*n* = 13)^a^	Nusinersen (*n* = 76)^b^	Onasemnogene abeparvovec (*n* = 14)^c^	All patients (*N* = 174)
Age at screening, years, median (range)	16 (11–36)	30 (16–58)	12 (1–60)	2 (1–5)	14 (1–60)
> 18 years, *n* (%)	31 (44)	11 (85)	21 (28)	0	63 (36)
< 2 years, *n* (%)	0	0	3 (4)	3 (21)	6 (3)
Age of onset of initial symptoms, months, median (range)	13 (0–258)	9 (0–256)	12 (0–188)	7.8 (0–10)	12 (0–258)
Time between onset of initial SMA symptoms and first treatment, months, median (range)	186.1 (28–397)	268.7 (188–702)	134.5 (18–719)	29 (20–59)	166.1 (18–719)
Sex, female, *n* (%)	36 (51)	4 (31)	36 (47)	3 (21)	79 (45)
SMA type, *n* (%)					
1	2 (3)	0	9 (12)	4 (29)	15 (9)
2	50 (70)	5 (39)	43 (57)	10 (71)	108 (62)
3	19 (27)	8 (62)	24 (32)	0	51 (29)
*SMN2* copy number, *n* (%)					
1	0	0	0	3 (21)	3 (2)
2	1 (1)	1 (8)	10 (13)	1 (7)	13 (8)
3	65 (92)	6 (46)	55 (72)	10 (71)	136 (78)
4	5 (7)	6 (46)	11 (15)	0	22 (13)
Motor function at baseline, *n* (%)^d^					
Non-sitters	29 (41)	7 (54)	21 (28)^e^	2 (14)^e^	59 (34)^e^
Sitters	42 (59)	3 (23)	43 (57)^e^	12 (86)^e^	100 (57)^e^
Walkers	0	3 (23)	12 (16)	0	15 (9)
Pulmonary care (invasive or non-invasive)^f^					
Yes	39 (55)	2 (15)	43 (57)^g^	10 (71)^g^	87 (52)^g^
Missing	0	1 (8)	0	0	1(1)
Baseline HFMSE total score < 10, *n* (%)					
Yes	59 (83)	8 (62)	35 (48)^g,h^	3 (27)^g,i^	105 (63)^g,j^
Scoliosis, *n* (%)					
Yes	66 (93)	9 (69)	61 (84)^g,h^	3 (27)^g,i^	139 (83)^g,j^
> 40° curvature	36 (51)	3 (23)	27 (37)^g,h^	0	66 (39)^g,i^
Scoliosis surgery, *n* (%)					
Yes	53 (75)	2 (15)	22 (30)^g^	0^g^	80 (48)^g^
Hip subluxation or dislocation, *n* (%)					
Yes	20 (28)	2 (15)	25 (34)^g,h^	4 (36)^g,i^	51 (30)^g,i^

CCOD: Jan 31, 2022. Intent-to-treat patients

*CCOD *clinical cut-off date; *HFMSE *Hammersmith Functional Motor Scale—Expanded, *HINE-2* Hammersmith Infant Neurological Examination, Module 2, *MFM* Motor Function Measure, *SMA* spinal muscular atrophy, *SMN* survival of motor neuron

^a^All but three patients enrolled in JEWELFISH were non‑treatment-naïve; these three patients who were previously enrolled in the MOONFISH trial were treatment-naïve as they received placebo and were never switched to RG7800 treatment

^b^Three patients in the nusinersen group had also received olesoxime (NCT01302600) previously

^c^One patient in the onasemnogene abeparvovec group received treatment with onasemnogene abeparvovec first followed by nusinersen. Ten patients were enrolled in STRONG (NCT03381729), three patients in STR1VE (NCT03306277) and one patient in STR1VE-EU (NCT03461289) prior to enrollment in JEWELFISH

^d^Non-sitters are defined as scoring 0 on Item 9 of the MFM whereas sitters scored ≥ 1 on Item 9 of the MFM but did not qualify as ambulant. Ambulant patients are defined as walkers

^e^For patients aged < 2 years, baseline motor milestones were evaluated by the HINE-2

^f^Use of pulmonary care in the 2 weeks prior to baseline assessment

^g^Only reported for patients aged 2–60 years

^h^*n* = 73

^i^*n* = 11

^j^*n* = 168

### Safety

The median duration of exposure to risdiplam was 26.8 months (range 0.9–59) and the median dose intensity (i.e., the number of doses received divided by the expected number of doses) of risdiplam in all patients was 99.7% (range 24.7–100%). Safety results in all patients enrolled in JEWELFISH (*n* = 174) are summarized in Table 2. Overall, 96% of patients reported ≥ 1 AE, and 20% of patients reported ≥ 1 SAE. In patients previously treated with nusinersen, 96% reported ≥ 1 AE and 21% ≥ 1 SAE. All patients previously treated with onasemnogene abeparvovec experienced ≥ 1 AE, whereas 29% had ≥ 1 SAE. There was one SAE (Grade 2 supraventricular tachycardia) that was considered related to risdiplam treatment in a patient previously treated with olesoxime. There were no treatment-related SAEs in any patients who were previously enrolled in the MOONFISH study or who had previously received treatment with nusinersen or onasemnogene abeparvovec.

**Table 2:** AEs observed in the JEWELFISH study over 24 months of treatment

	Previous treatment				
	Olesoxime (*n* = 71)	MOONFISH study (RG7800) (*n* = 13)^a^	Nusinersen (*n* = 76)	Onasemnogene abeparvovec (*n* = 14)	All patients (*N* = 174)^b^
Total PY at risk	155.6	41.8	170.6	29.1	397.1
Patients with ≥ 1 AE, *n* (%)	67 (96)	12 (92)	73 (96)	14 (100)	166 (96)
Total number of AEs	508	84	641	96	1329
Overall rate of AEs, per 100 PY (95% CI)	326.43 (298.65–356.09)	201.09 (160.40–248.97)	375.72 (347.19–405.96)	329.70 (267.06–402.62)	334.66 (316.91–353.15)
Total number of SAEs	19	3	47	7	76
Overall rate of SAEs, per 100 PY (95% CI)	12.21 (7.35–19.07)	7.18 (1.48–20.99)	27.55 (20.24–36.63)	24.04 (9.67–49.53)	19.14 (15.08–23.95)
Total number of deaths	0	0	0	0	0
Total number of patients with ≥ 1, *n* (%)					
SAE	12 (17)	3 (23)	16 (21)	4 (29)	35 (20)
Treatment-related SAE	1 (1)^c^	0	0	0	1 (1)^c^
SAE leading to dose modification/interruption	4 (6)	1 (8)	4 (5)	1(7)	10 (6)
AE leading to withdrawal from treatment	0	0	1 (1)^d^	0	1 (1)^d^
Treatment-related AE	12 (17)	6 (46)	20 (26)	0	38 (22)
Treatment-related AE leading to withdrawal from treatment	0	0	0	0	0
Most common AEs^e^, *n* (number of patients [%])					
Pyrexia	11 (16)	2 (15)	23 (30)	6 (43)	42 (24)
URTI	16 (23)	0	17 (22)	4 (29)	37 (21)
Headache	13 (19)	1(8)	17 (22)	0	31 (18)
Nasopharyngitis	8 (11)	2 (15)	12 (16)	5 (36)	27 (16)
Diarrhea	6 (9)	0	17 (22)	1 (7)	24 (14)
Nausea	7 (10)	0	14 (18)	1 (7)	22 (13)
Cough	6 (9)	0	12 (16)	3 (21)	21 (12)
Most common SAEs^f^, *n* (number of patients [%])					
Pneumonia	1 (1)	0	3 (4)	1 (7)	5 (3)
Respiratory failure	1 (1)	0	3 (4)	0	4 (2)
Respiratory distress	0	0	2 (3)	1 (7)	3 (2)
LRTI	2 (3)	0	1 (1)	0	3 (2)
URTI	0	0	3 (4)	0	3 (2)

CCOD: Jan 31, 2022. As follow-up duration is different between groups, the overall rate of AEs and SAEs cannot be compared. Multiple occurrences of the same AE in one individual are counted only once except for the “Total number of AEs” row, for which multiple occurrences of the same AE are counted separately

*AE* adverse event, *CCOD* clinical cut-off date, *CI* confidence interval, *LRTI* lower respiratory tract infection, *PY* patient-years, *SAE* serious AE, *URTI* upper respiratory tract infection

^a^All but three patients enrolled in JEWELFISH were non‑treatment-naïve; these three patients who were previously enrolled in the MOONFISH trial were treatment-naïve as they received placebo and were never switched to RG7800 treatment

^b^One patient withdrew from the study at baseline; therefore, 173 patients received risdiplam. Risdiplam treatment duration for all patients in months, median (range): 26.8 (0.9–59)

^c^An SAE of supraventricular tachycardia was considered related to risdiplam treatment by the investigator (in the context of hypoxia) and resolved with ongoing treatment with risdiplam

^d^Irritable bowel syndrome and panic attack, which were unrelated to risdiplam, led to the withdrawal of one patient who was previously treated with nusinersen

^e^AEs reported in ≥ 12% of all patients

^f^SAEs reported in > 2% of all patients. Includes AEs with onset from the first dose of the study drug up to the CCOD

The proportion of patients with Grade 3–5 AEs in the overall safety population was 21%. When separated by patients previously treated with nusinersen or onasemnogene abeparvovec, the proportion was 25% and 21%, respectively. Six Grade 4 AEs occurred in three patients, which were unrelated to risdiplam and resolved without dose modification, and there were no patients with Grade 5 AEs.

Across all patients, the most common AEs were pyrexia (24%), upper respiratory tract infection (URTI, 21%), and headache (18%). Patients previously treated with nusinersen experienced pyrexia (30%), diarrhea, headache, and URTI (22% for each), whereas patients previously treated with onasemnogene abeparvovec experienced pyrexia (43%), nasopharyngitis (36%), and URTI (29%) as common AEs. Pneumonia (3%) was the most reported SAE for all JEWELFISH patients, including those previously treated with nusinersen (4%) or onasemnogene abeparvovec (7%).

**Fig. 2:**
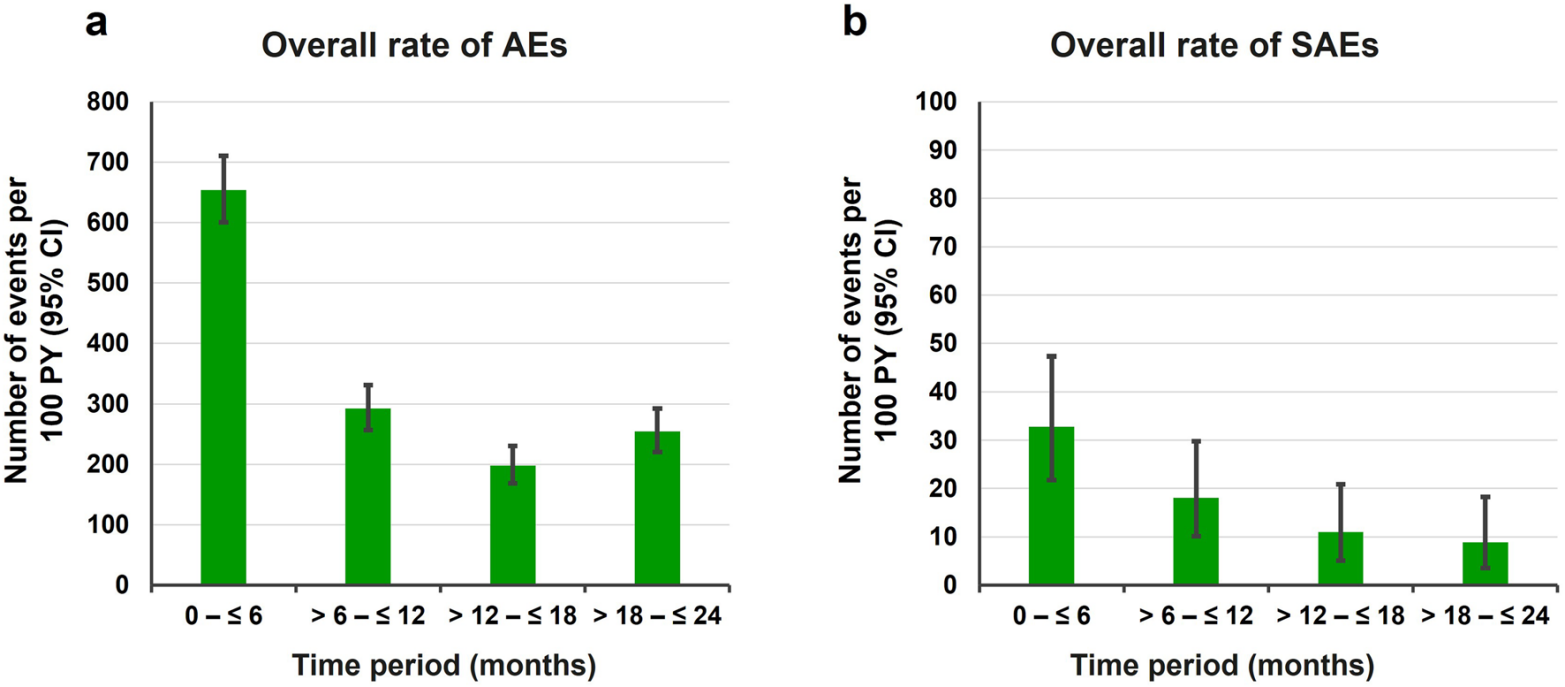
Rates of AEs over time in JEWELFISH. **a** Overall rate of AEs over time, **b** Overall rate of SAEs over time. *AE* adverse event, *CI* confidence interval, *PY* patient-years, *SAE* serious AE

When adjusted for patient-years (PY) at risk, the overall AE and SAE rates for all patients during the total treatment period were 334.66 events per 100 PY (95% confidence interval [CI] 316.91–353.15) and 19.14 events per 100 PY (95% CI 15.08–23.95), respectively. The rate of AEs and SAEs decreased over the duration of the JEWELFISH study (Fig. 2). During the first 6 months of treatment, the AE rate was 653.86 events per 100 PY (95% CI 600.77–710.38; total PY at risk: 85.5 years) and remained stable for the following three 6-month periods. Over the period of 0–12 months of treatment, the AE rate was 475.51 events per 100 PY (95% CI 443.17–509.59; total PY at risk: 168.7 years) and the SAE rate was 25.50 events per PY (95% CI 18.45–34.34). Comparing the 0–12 months treatment period with the 12–24 months treatment period, a 50% decrease in the rate of AEs was observed, with 225.65 AEs per 100 PY (95% CI 203.04–250.10; total PY at risk: 160.9 years) and 9.95 SAEs per 100 PY (95% CI 5.69–16.15).

The decline in the overall rate of AEs was reflected in decreases in the rates of the most common AEs. Comparing the rates of the common AEs per 100 PY between the first and fourth 6-month treatment periods (0 to ≤ 6 months vs. > 18 to ≤ 24 months) showed declines in pyrexia (31.58 to 13.93), URTI (37.43 to 13.93), and headache (44.45 to 7.60); the rate of the most common SAE of pneumonia also decreased (4.68 to 1.27). There were no treatment-related safety findings that led to a withdrawal.

A review of all available safety laboratory results, vital signs, electrocardiograms, and ophthalmologic assessments did not show any clinically significant adverse findings compared with baseline. As of the CCOD, there have been no safety findings in patients reflective of potential risks previously identified from non-clinical toxicology studies (effects on epithelial tissues, retinal toxicity, or hematologic effects).

### Secondary endpoint

#### PK/PD of risdiplam

Risdiplam exposure was comparable across the JEWELFISH patient population at the selected dosing regimen and the mean area under the concentration–time curve from 0–24 h (AUC_0–24_) was 1700 ng.h/mL. Details of the PK/PD from JEWELFISH will be reported as part of a full analysis of the PK/PD across the risdiplam clinical trial program.

**Fig. 3:**
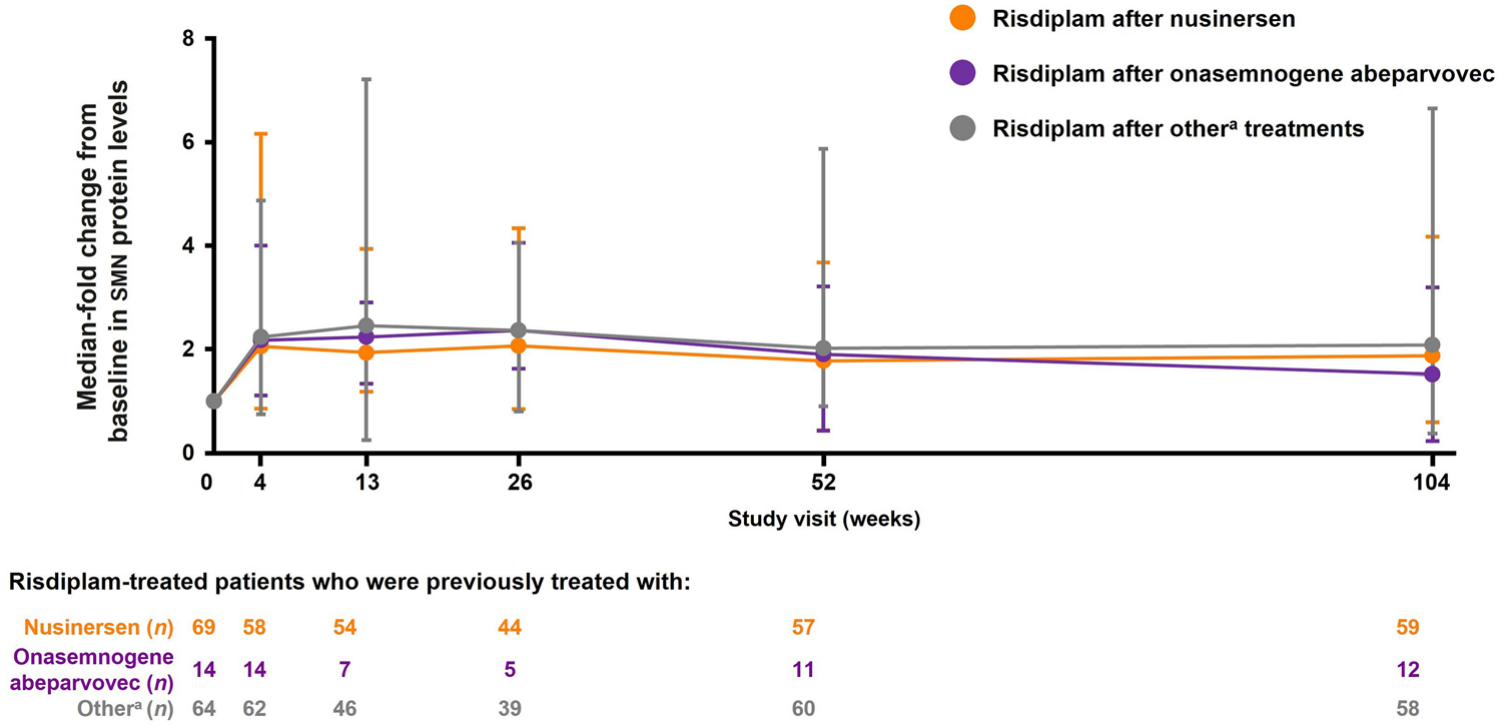
Median-fold change from baseline in SMN protein level in blood by previous treatment *SMN* survival of motor neuron. ^a^Patients previously enrolled in MOONFISH or treated with olesoxime. Error bars represent range (minimum–maximum values)

At baseline, the concentration of SMN protein in blood was comparable across all treatment groups. After 4 weeks of treatment with risdiplam, SMN protein levels in blood showed a two-fold change from baseline in all groups, regardless of previous treatment, and sustained increases in the level of SMN protein in blood were observed over 24 months of treatment (Fig. 3).

At month 24, the median fold (minimum–maximum) change in SMN protein levels in whole blood was 1.87 (0.60–4.17) and 1.52 (0.23–3.19) in patients previously treated with nusinersen and onasemnogene abeparvovec, respectively.

### Key exploratory efficacy endpoints

#### Motor function in patients aged 2–60 years

**Fig. 4:**
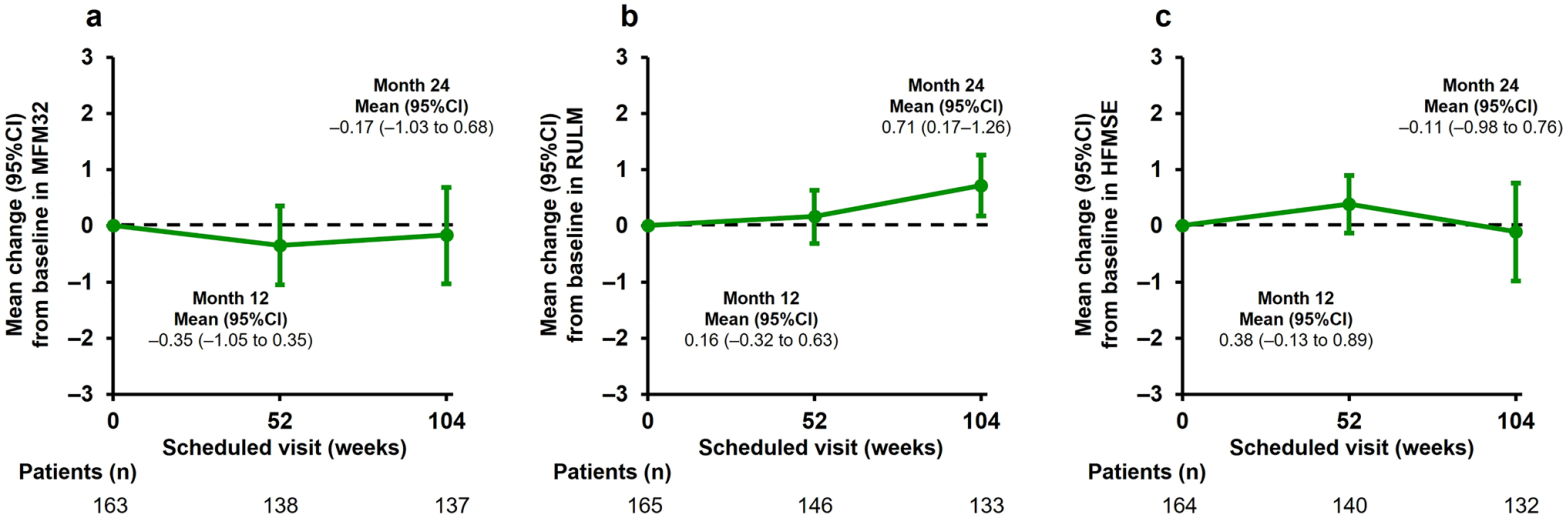
Mean change from baseline in motor function assessments in patients aged 2–60 years. **a** Mean change from baseline in MFM32 total score, **b** Mean change from baseline in RULM total score, **c** Mean change from baseline in HFMSE total score. *CI* confidence interval, *HFMSE* Hammersmith Functional Motor Scale—Expanded, *MFM32* 32-item Motor Function Measure, *RULM* Revised Upper Limb Module

Exploratory motor function outcomes (assessed by the MFM32, RULM, and HFMSE scales) were analyzed from the ITT population, which included 167 patients aged 2–60 years (Fig. 4a-c, Table 3). Stabilization of the MFM32 total score was observed from baseline to month 24 (Fig. 4a), with a mean change of –0.17 (95% CI –1.03 to 0.68, *n* = 137).

**Table 3:** Exploratory efficacy motor function endpoints (patients aged 2–60 years)

	All patients (*N* = 167)		
	Baseline	Month 12	Month 24
MFM32			
Mean total score (95% CI) *n*	39.93 (37.07–42.80) 163	40.41 (37.30–43.52) 142	40.67 (37.60–43.74) 140
Mean change from baseline in total score (95% CI) *n*	–	–0.35 (–1.05 to 0.35) 138	–0.17 (–1.03 to 0.68) 137
RULM			
Mean total score (95% CI) *n*	16.60 (15.19–18.01) 165	16.48 (14.89–18.07) 149	17.49 (15.86–19.12) 136
Mean change from baseline in total score (95% CI) *n*	–	0.16 (–0.32 to 0.63) 146	0.71 (0.17–1.26) 133
HFMSE			
Mean total score (95% CI) *n*	12.13 (9.86–14.41) 164	12.83 (10.27–15.39) 142	12.71 (10.02–15.39) 134
Mean change from baseline in total score (95% CI) *n*	–	0.38 (–0.13 to 0.89) 140	–0.11 (–0.98 to 0.76) 132

	Ambulant patients aged 6–60 years (*n* = 11)		
6MWT^a^			
Mean total distance walked, m (95% CI) *n*	165.89 (86.13–245.65) 9	187.44 (86.24–288.64) 9	209.13 (100.44–317.81) 8
Mean change from baseline in total distance walked, m (95% CI) *n*	–	21.56 (–13.96 to 57.07) 9	30.88 (–5.54 to 67.29) 8

*6MWT* 6-min walk test, *CI* confidence interval, *HFMSE* Hammersmith Functional Motor Scale—Expanded, *MFM32* 32-item Motor Function Measure, *RULM* Revised Upper Limb Module

^a^6MWT was added as the exploratory endpoint for ambulant patients aged 6–60 years after the start of the study and therefore not all patients have baseline measurements, only patients with a baseline visit are included here

Stabilization in upper limb function as shown by the mean RULM total score was also observed from baseline to month 24 (Fig. 4b, Table 3). At month 24, the mean change from baseline in RULM total score was 0.71 (95% CI 0.17–1.26, *n* = 133). On average, the HFMSE total score was stable at month 24 compared with baseline (Fig. 4c, Table 3). At month 24, the mean change from baseline in the HFMSE total score was –0.11 (95% CI –0.98 to 0.76, *n* = 132). Exploratory motor function endpoints (MFM32, RULM, and HFMSE) at month 12 and month 24 are reported in Online Resource 3 (Table S1).

An increase in the caregiver-reported SMAIS–ULM total score was observed from baseline to month 24. At month 24, the mean change from baseline in caregiver-reported SMAIS–ULM total score was 1.90 (95% CI 0.70–3.10, *n* = 110). Respiratory function, as measured by the sniff nasal inspiratory pressure test, showed a mean change of 0.14% (95% CI –2.64 to 2.92, *n* = 113) from baseline to month 24. Full details on the SMAIS–ULM and respiratory function can be found in Online Resource 3.

A post hoc analysis of motor function measures (MFM32, RULM, and HFMSE) among a subgroup of patients aged 25–60 years was also performed. Stabilization after 24 months of treatment with risdiplam was observed within this age group (Table S2).

#### Analyses of motor function measures by previous treatment

Exploratory motor function measures following 24 months of treatment with risdiplam are reported for nusinersen (*n* = 59) and onasemnogene abeparvovec patients (*n* = 9) aged 2–60 years (Table S1). For patients who were previously treated with nusinersen, the mean (95% CI) change from baseline to month 24 in the MFM32 total score was –1.25 (–2.55 to 0.04, *n* = 59); the change in RULM total score was 0.50 (–0.20 to 1.20, *n* = 58); and the change in HFMSE total score was –1.21 (–2.79 to 0.36, *n* = 56).

For patients who had previously received onasemnogene abeparvovec, an increase from baseline to month 24 was observed in each of the motor function scales: for the MFM32 total score, the mean (95% CI) change was 6.25 (0.31–12.19, *n* = 9); for the RULM total score, the change was 6.11 (2.11–10.11, *n* = 9); and for the HFMSE total score, the change was 7.11 (3.93–10.29, *n* = 9).

#### 6MWT in ambulant patients

Overall, 15 patients with Type 3 SMA were ambulant at baseline. Of the 14 ambulant patients aged ≥ 6 years, 13 patients were assessed with the 6MWT at any time point during the study. All 13 ambulant patients maintained their ability to walk. Among the eight patients who were evaluated both at baseline and at month 24 (all eight patients were previously treated with nusinersen) there was a mean (95% CI) increase from baseline in the total distance walked of 30.88 m (–5.54 to 67.29, *n* = 8) at month 24 (Table S1).

The two ambulant patients who did not perform the 6MWT were assessed with regard to their ability to walk using the HFMSE (achieving a score of 2 points on Item 20, ‘stepping’). These two patients scored 2 on all assessments. Therefore, all ambulant patients maintained their ability to walk.

#### Motor function in patients aged < 2 years

The ITT population included six patients aged < 2 years, three had previously received nusinersen and three had previously received onasemnogene abeparvovec (Table 1). Sitting for 5 s (Item 22 of the BSID-III Gross Motor Scale) was present at baseline in one (33.3%) patient previously treated with nusinersen, and in two patients (66.6%) previously treated with onasemnogene abeparvovec; by month 24, all six patients (100%) were sitting for 5 s.

Sitting without support for ≥ 30 s (Item 26 of the BSID-III Gross Motor Scale) was present in two patients (33%) at baseline; by month 24, three patients (50%) had achieved the milestone.

At month 24 of treatment, all six patients (100%) were classified as motor milestone responders. An infant was classified as a motor milestone responder if more motor milestones on the HINE-2 showed improvement than showed worsening compared with baseline.

### Post hoc analyses of safety data

#### Comparison between non-treatment-naïve and treatment-naïve patients

**Fig. 5:**
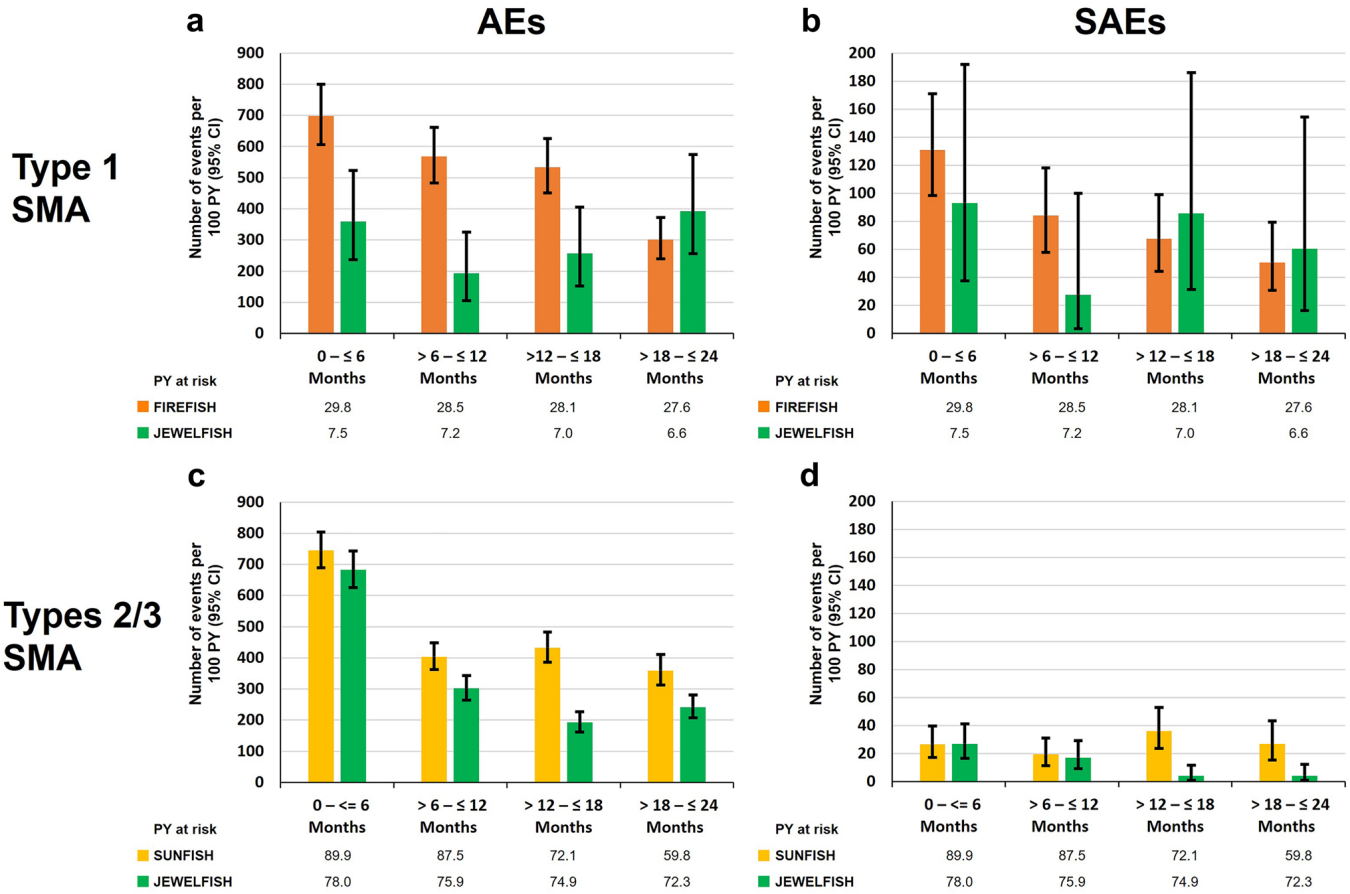
Rates of AEs and SAEs in non-treatment-naïve patients in JEWELFISH and treatment-naïve patients (FIREFISH, Type 1 SMA and SUNFISH, Types 2/3 SMA) treated with risdiplam. **a** AE and **b** SAE rates in treatment-naïve patients with Type 1 SMA in FIREFISH (*N* = 62) and non-treatment-naïve patients with Type 1 SMA in JEWELFISH (*N* = 15), **c** AE and **d** SAE rates in treatment-naïve patients with Types 2/3 SMA in SUNFISH (*N* = 179) and non-treatment-naïve patients with Types 2/3 SMA in JEWELFISH (*N* = 158). *AE* adverse event, *CI* confidence interval, *PY* patient-years, *SAE* serious AE, *SMA* spinal muscular atrophy

The rates of AEs and SAEs in patients in the JEWELFISH study were compared with the rates observed in treatment-naïve patients in the FIREFISH [22, 23] and SUNFISH [24] trials based upon SMA type (Fig. 5). In patients with Type 1 SMA in JEWELFISH, the overall AE rate was 323.40 events per 100 PY (95% CI 263.69–392.58) versus 465.53 events per 100 PY (95% CI 436.20–496.39) in the previously treatment-naïve patients with Type 1 SMA treated with risdiplam in the FIREFISH study. In patients with Types 2 and 3 SMA treated with risdiplam in JEWELFISH, the overall AE rate was 335.63 events per 100 PY (95% CI 317.11–354.95) versus 542.31 events per 100 PY (95% CI 513.00–572.87) in previously treatment-naïve patients with Types 2 and 3 SMA treated with risdiplam in the SUNFISH Part 2 study.

A decline in the rates of AEs and SAEs was observed for both non-treatment-naïve and treatment-naïve patients in the risdiplam pivotal trials over a 24-month period (Fig. 5).

## Discussion

This analysis of the JEWELFISH study examined the safety of risdiplam after 24 months of treatment in patients who had previously received an SMA DMT or were previously enrolled in a trial of an investigational therapy for SMA. Based on the review of all available safety data, risdiplam was well tolerated and the safety profile was in accordance with the known safety profile of risdiplam irrespective of previous treatment. The safety profile of risdiplam in JEWELFISH is consistent with results from treatment-naïve individuals who received risdiplam in the FIREFISH and SUNFISH studies.

The majority of AEs were reflective of underlying disease, and most AEs were mild to moderate in intensity. There was a considerable decline in the AE rate after the first 6 months of treatment and a decrease in the overall rate of SAEs per 100 PY over time. More specifically, the AE rate declined by over 50% between the first and second years of risdiplam treatment, and this decrease was reflected in the most common AEs (pyrexia, URTI, and headache). No treatment-related safety findings have led to withdrawal from treatment.

Risdiplam treatment led to a median two-fold increase in blood SMN protein levels after 4 weeks of treatment and increased SMN protein was observed for the 24 months of follow-up, irrespective of previous treatment.

Following 24 months of risdiplam treatment in this weak patient population, the exploratory efficacy data show stabilization in motor function as assessed by the MFM32, RULM, and HFMSE. Although direct comparisons with published data cannot be made, longitudinal studies of untreated patients consistently report a considerable decline in measures of motor function [25–29]. For instance, natural history studies evaluating untreated patients with Types 2 and 3 SMA have reported a decline in mean MFM32 total score over 24 months (–2.08 points; patients aged 2–30 years) [26], in median RULM total score over 24 months (–0.79 points; patients aged 5–56 years) [27], and mean HFMSE total score over 24 months (–1.74 points; patients aged 2–34 years) [29]. A survey that captures patient expectations at the time when DMTs were made available, identified that stabilization of clinical condition is a benefit [30].

Among ambulant patients, an increase in the total distance walked in the 6MWT was observed after 24 months of risdiplam treatment. In a natural history study of patients of a similar age, there was a decline in the total distance walked in the 6MWT (–20.8 m per year, patients 11–19 years of age) [31]. None of the ambulant patients assessed by the 6MWT lost their ability to walk.

Overall, the coronavirus disease 2019 (COVID-19) pandemic did not significantly impact the ability to monitor and manage patient safety during the study, and treatment compliance, as reflected by the dose intensity, was high across all patients (99.7% of planned doses were received). The oral dosing regimen reduced the need for travel to the hospital to receive treatment and was particularly beneficial when participants may have been limited by travel restrictions. For many patients who enrolled towards the end of the recruitment period, the 6-month visit fell during the first peak of the pandemic and so attendance to the clinical sites was affected due to restriction measures at site and national levels. On-site visit attendance improved after the COVID-19 travel restrictions were relaxed, as demonstrated by the high percentage of patients who completed visits at month 12 and month 24.

### Limitations

JEWELFISH was an open-label study; there was no control group and all efficacy endpoints were exploratory. Therefore, caution should be taken in making conclusions from the efficacy data.

Potentially, the broad inclusion criteria of JEWELFISH, which enrolled patients who had previously received other SMA treatments, may have been biased towards a patient population that had not optimally responded to previous treatment and/or who subjectively felt they needed more treatment efficacy.

Furthermore, caution should be taken when interpreting the HFMSE score in weak patients due to floor effects [32], and for the JEWELFISH population, other motor function scores such as the MFM32 or RULM may be more informative. Caution should be also taken with the interpretation of the subgroup data analyses which were conducted with a small sample of patients. Also, differences in the demographics between the subgroups, in particular, those previously treated with nusinersen or onasemnogene abeparvovec, make comparisons of efficacy difficult to interpret. There is a lack of a single universal functional outcome that can sensitively measure functional changes among this broad and heterogeneous patient group, thus making the interpretation of efficacy challenging.

Some assessments may have been affected due to travel restrictions during the COVID-19 pandemic. Although these restrictions may have contributed to lower rates of AEs between months 6 and 12 of the study, when patients were required to spend more time indoors with limited in-person socialization, the exact impact cannot be determined.

## Conclusions

The safety profile of risdiplam in the JEWELFISH study was consistent with results from the FIREFISH [22, 23] and SUNFISH studies [24]. A similar PD response was observed in this patient population; a median two-fold increase in SMN protein levels was achieved across all subgroups regardless of prior treatment, and the increase was consistent with results observed in treatment-naïve patients from the FIREFISH and SUNFISH trials. Although efficacy endpoints were exploratory, overall stabilization of motor function was observed in treated patients from baseline to month 24. All ambulant patients retained their ability to walk.

Results from the analysis of JEWELFISH after 24 months provide further evidence that risdiplam can be safely received by patients who have previously been treated with nusinersen or onasemnogene abeparvovec. The JEWELFISH open-label extension phase is ongoing and will provide further long-term safety data on risdiplam in this non-treatment-naïve patient population.

### Supplementary Information

Below is the link to the electronic supplementary material.

Supplementary file1 (PDF 1913 KB)

Supplementary file3 (PDF 1407 KB)

Supplementary file2 (DOCX 53 KB)

## Data Availability

For up-to-date details on Roche's Global Policy on the Sharing of Clinical Information and how to request access to related clinical study documents, see here: https://go.roche.com/data_sharing. Anonymized records for individual patients across more than one data source external to Roche cannot, and should not, be linked due to a potential increase in risk of patient re-identification.
