# Supplementary material for: JEWELFISH: 24-month results from an open-label study in non-treatment-naïve patients with SMA receiving treatment with risdiplam
Source: J Neurol. 2024 May 11;271(8):4871–84. doi: 10.1007/s00415-024-12318-z (PMC11319388; doi:10.1007/s00415-024-12318-z)

PLEASE READ CAREFULLY:

By opening the attached document you agree to the following terms of use:

- You may not use this document or the information contained herein to a regulatory authority in connection with an application for a marketing authorization or any other regulatory submission without the express written consent of Roche. Please contact global.data_sharing@roche.com

- You may not use this document or the information contained herein to identify clinical trial patients.

- You may not copy, reproduce, or make this document available in any manner that would permit a third-party to review or use the document without first agreeing to these terms of use.

To open the document, please follow the instructions below:

- Please use Adobe Acrobat software to view the document.

- On the right hand panel, open the Attachments tab (paper clip) to see the document attached *(see screenshot below for an example).*

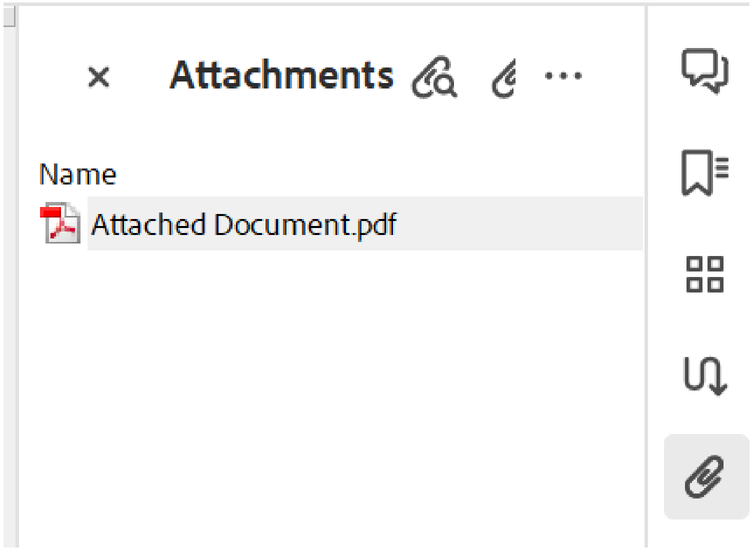

Supplement: Supplementary file 1 — Supplementary file1 (PDF 1913 KB) [file 415_2024_12318_MOESM1_ESM.pdf]
