# Supplementary material for: JEWELFISH: 24-month results from an open-label study in non-treatment-naïve patients with SMA receiving treatment with risdiplam
Source: J Neurol. 2024 May 11;271(8):4871–84. doi: 10.1007/s00415-024-12318-z (PMC11319388; doi:10.1007/s00415-024-12318-z)
Supplement: Supplementary file 3 — Supplementary file2 (DOCX 53 KB) [file 415_2024_12318_MOESM3_ESM.docx]

**JEWELFISH: 24-month results from an open-label study in non-treatment-naïve patients with SMA receiving treatment with risdiplam**

Claudia A. Chiriboga^1^*, Claudio Bruno^2,3^, Tina Duong^4^, Dirk Fischer^5^, Eugenio Mercuri^6^, Janbernd Kirschner^7^, Anna Kostera-Pruszczyk^8^, Birgit Jaber^9^, Ksenija Gorni^10^, Heidemarie Kletzl^11^, Imogen Carruthers^12†^, Carmen Martin^12^, Renata S. Scalco^13^, Paulo Fontoura^10^, Francesco Muntoni^14^, on behalf of the JEWELFISH Study Group

1. Department of Neurology, Columbia University Irving Medical Center, New York, NY, USA
2. Centre of Translational and Experimental Myology, IRCCS Istituto Giannina Gaslini, Genoa, Italy
3. Department of Neuroscience, Rehabilitation, Ophthalmology, Genetics, Maternal and Child Health-DINOGMI, University of Genoa, Genoa, Italy
4. Department of Neurology, Stanford University, Palo Alto, CA, USA
5. Division of Neuropediatrics, University Children's Hospital Basel, University of Basel, Basel, Switzerland
6. Pediatric Neurology Institute, Catholic University and Nemo Pediatrico, Fondazione Policlinico Gemelli IRCCS, Rome, Italy
7. Department of Neuropediatrics and Muscle Disorders, Medical Center-University of Freiburg, Faculty of Medicine, Freiburg, Germany
8. Department of Neurology, Medical University of Warsaw, Warsaw, Poland, ERN EURO-NMD
9. Pharma Development, Safety, F. Hoffmann-La Roche Ltd, Basel, Switzerland
10. PDMA Neuroscience and Rare Disease, F. Hoffmann-La Roche Ltd, Basel, Switzerland
11. Roche Pharmaceutical Research and Early Development, Roche Innovation Center Basel, Basel, Switzerland
12. Roche Products Ltd, Welwyn Garden City, UK
13. Product Development Neuroscience, F. Hoffmann-La Roche Ltd, Basel, Switzerland
14. The Dubowitz Neuromuscular Centre, NIHR Great Ormond Street Hospital Biomedical Research Centre, Great Ormond Street Institute of Child Health University College London, & Great Ormond Street Hospital Trust, London, UK.

^†^Employee of Roche Products Ltd at the time of data collection.

*Corresponding author: Claudia Chiriboga (email address: cac3@cumc.columbia.edu)

**Supplementary material**

**JEWELFISH Study Group**

**JEWELFISH Principal Investigators (in bold) and site study personnel**

*Belgium*: **Nicolas Deconinck, M.D.**; Ophthalmologists: Irina Balikova, M.D., Inge Joniau, M.D., Physiotherapists: Valentine Tahon, Sylvia Wittevrongel; **Nathalie Goemans, M.D.**; Ophthalmologists: Catherine Cassiman, Lies Prove; Physiotherapists: Lisa Vancampenhout, Marleen van den Hauwe, Annelies Van Impe; *France*: **Claude Cances,** **M.D.**; Ophthalmologists: Vincent Soler, Lauriane Maillard De La Morandais; Orthoptist: Delphine Vovan; Co-investigator: Pascal Cintas; Clinical research coordinator: Françoise Auriol: Study Coordinator: Marianne Mus, Gwennaelle Alphonsa; Physiotherapist: Valerie Bellio, Olaia Gil Mato; **Jean-Baptiste Davion, M.D.**; Sub-investigator: Florence Flamein; Study nurse: Cécile Evrard; Study coordinator: Amina Ziouche; Ophthalmologists: Ikram Bouacha-Allou, Philippe Debruyne, Gilles Derlyn, Sabine Defoort; Florian Leroy, Loïc Danjoux; **Isabelle Desguerre, M.D.**; Ophthalmologists: Dominique Bremond-Gignac, Maxence Rateuax; Physiotherapist: Elodie Deladrière; **Carole Vuillerot, M.D.**; Study Coordinator: Quentin Veillerot; Ophthalmologist: Bénédicte Sibille-Dabadi; Physiotherapists: Aurélie Barrière, Marie Tinat; Study coordinator: Manel Saidi; Sub-investigators: Stephanie Fontaine, Camille De Montferrand, Laure Le-Goff, Aurélie Portefaix; **Ulrike Walther Louvier, M.D.**; Ophthalmologists: Pierre-André Duval, Pascale Caradec; Physiotherapists: Souad Touati, Alberto Zamora Herranz; *Germany:* **Janbernd Kirschner, M.D.**; Ophthalmologist: Jan Bollig, Fanni Molnar; Physiotherapist: Sibylle Vogt; Principal Investigator: Astrid Pechmann; Deputy: David Schorling; Study coordinator: Sabine Wider; **Heike Kölbel, M.D.**; Sub investigators: Ulrike Schara, Frederik Braun, Andrea Gangfuss; Deputy: Tim Hagenacker; Ophthalmologists: Anja Eckstein, Dirk Dekowski, Michael Oeverhaus, Mareile Stoehr; Physiotherapist: Barbara Andres; Site coordinator: Karin Smuda; *Italy:* **Enrico Bertini, M.D.**; Site investigator: Adele D’Amico; Ophthalmologists: Sergio Petroni, Paola Valente; Physiotherapists: Anna Maria Bonetti, Adelina Carlesi, Irene Mizzoni; **Claudio Bruno, M.D.**; Site investigators: Marina Pedemonte, Noemi Brolatti; Ophthalmologists: Enrico Priolo, Giuseppe Rao, Lorenza Sposetti; Physiotherapist: Simone Morando; **Giacomo Comi, M.D.**; Ophthalmologists: Silvia Osnaghi, Valeria Minorini; Physiotherapists: Francesca Abbati, Federica Fassini, Michaela Foà, Maria Amalia Lopopolo; Neurologists: Francesca Magri, Alessandra Govoni, Megi Meneri; Biologist:Valeria Parente; **Eugenio Mercuri, M.D.**; Clinicians: Laura Antonaci, M.D.; Maria Carmela Pera, M.D.; Marika Pane, M.D.; Ophthalmologists: Giulia Maria Amorelli, Costanza Barresi, Guglielmo D’Amico, Lorenzo Orazi; Physiotherapists: Giorgia Coratti; Roberto De Sanctis; **Giuseppe Vita, M.D.**; Site investigator: Maria Sframeli; Gian Luca Vita; Ophthalmologists: Pasquale Aragona, Leandro Inferrera, Elisa Imelde Postorino; Orthopist; Daniela Montanini; Physiotherapists: Vincenzo Di Bella, Concetta Donato, Elisabetta Calà; *Netherlands:* **Ludo Van der Pol MD;** Optic technician: Jos Aalbers; Ophthalmologists: Joke de Boer, Saskia Imhof; Orthoptist: Pascale Cooijmans; Pediatric physiotherapists: Thijs Ruyten, Danny Van Der Woude; *Poland:* **Anna Kostera-Pruszczyk, M.D.**; Ophthalmologists: Beata Klimaszewska, Dominika Romańczak; Physiotherapists: Malgorzata Burlewicz, Zuzanna Gierlak-Wójcicka, Malwina Kępa, Adam Sikorski, Marcin Sobieraj, Jaroslaw Steinhagen; Neurologists: Anna Lusakowska, Biruta Kierdaszuk, Karolina Czeczko; *Switzerland:* **Dirk Fischer, M.D.**; Site investigator: Bettina Henzi; Ophthalmologists: Konstantin Gugleta, Akos Kusnyerik, Patricia Siems; Physiotherapists: Sabina Akos, Nora Frei, Christine Seppi, Christine Wondrusch Haschke; *United Kingdom:* **Michela Guglieri, M.D.**; **Volker Straub, M.D.**; Ophthalmologists: Richard Bell, Mahmoud Nassar, Stuart Page, Michael Patrick Clarke, Aedheen Regan; Physiotherapists; Anna Mayhew, Robert Muni Lofra; **Deepak Parasuraman, M.D.**; Ophthalmologists: Simone Bruschi, Abdul-Jabbar Ghauri; Orthoptist: Andrew Castle; Photographers: Saima Naqvi, Nicola Patt; **Mariacristina Scoto, M.D.**; Site investigator: Federica Trucco; Ophthalmologists: Robert H Henderson, Roopen Kukadia, Will Moore; Physiotherapists: Evelin Milev, Catherine Rye, Victoria Selby, Amy Wolfe; *United States:* **Basil Darras, M.D.**; Ophthalmologists: Anna Maria Baglieri, Anne Fulton; Physiotherapists; Courtney Lucken, Elizabeth Maczek, Amy Pasternak; **Claudia A Chiriboga, M.D.**; Ophthalmologists: Steven Kane, M.D.; Ophthalmologist technicians: Ma Edylin M. Bautista, Eileen Frommer, Noelle Pensec; Physiotherapists: Rachel Salazar, Cara Yochai, Rafael Rodrigues-Torres, Manroop Chawla; **John Day, M.D.**; Ophthalmologist: Shannon Beres; Physiotherapists: Richard Gee, Sally Dunaway Young; **Richard Finkel, M.D.**; Aledie Navas Nazario, M.D.; Ophthalmologist: Airaj Fasiuddin; Physiotherapist: Julie A. Wells; Research nurses: Jennifer Wilson, Debbie Berry; Research Coordinator: Virgina Rizzo; Research Pharmacist: Julie Duke; Sub investigator: Migvis Monduy; Pharmacy technician: Jorge Collado

**Sponsor (Roche) personnel**

Teresa Gidaro, Dominik Kraus, Francis Warren

**Supplementary methods**

**Complete list of objectives and endpoints**

**Primary objectives**

- To evaluate the safety and tolerability of risdiplam
  - Incidence of adverse events (overall, by severity and by relationship to study medication)
  - Incidence of serious adverse events
  - Incidence of treatment discontinuations due to adverse events
  - Incidence of laboratory abnormalities
  - Incidence of electrocardiogram abnormalities
  - Incidence of vital sign abnormalities
  - Incidence of suicidal ideation or behavior on the Columbia-Suicide Severity Rating Scale (C-SSRS)
  - Incidence of clinically significant findings on ophthalmologic examination
  - Incidence of clinically significant findings on neurologic examination
  - Anthropometric examination, including weight, height, head, and chest circumference.
- To investigate the pharmacokinetics (PK) of risdiplam and metabolites as appropriate
  - Concentration per timepoint listed
  - Maximum concentration observed (C_max_)
  - Area under the concentration–time curve (AUC)
  - Concentration at the end of a dosing interval (C_trough_) to assess steady state
  - Other PK parameters as appropriate.

**Secondary objectives**

- To investigate the PK/pharmacodynamic (PD) relationship of risdiplam.
- The PD investigations will include analyses of survival of motor neuron (SMN) mRNA splice forms and SMN protein
  - SMN mRNA in blood
  - SMN protein levels in blood.

**Exploratory objectives**

- To evaluate the efficacy of treatment with risdiplam:
  - In terms of the proportion of patients who experience a pre-specified disease-related adverse event
    - Proportion of patients who experience at least one disease-related adverse event by month 12 and month 24
    - Number of disease-related adverse events per patient-year at month 12 and month 24
  - In terms of motor function as assessed through the following measures:
    - 32-item Motor Function Measure in patients aged 2–60 years
    - Hammersmith Functional Motor Scale–Expanded in patients aged 2–60 years
    - Revised Upper Limb Module in patients aged 2–60 years
    - 6-min walk test (6MWT) of walking capacity in ambulant patients aged 6–60 years
    - Bayley Scales of Infant and Toddler Development, third edition (aged 1 year to < 2 years)
    - Hammersmith Infant Neurological Examination, Module 2 (HINE-2) motor milestones (aged 1 year to < 2 years)
  - In terms of achievement of motor milestones as assessed through the HINE-2 (aged 1 year to < 2 years)
  - On respiratory function as assessed through the following measures:
    - Sniff nasal inspiratory pressure (SNIP) in patients aged 2–60 years
    - Forced vital capacity (FVC), forced expiratory volume in 1 second (FEV_1_), and peak cough flow (PCF) in patients aged 6–60 years
  - To evaluate time-matched QT profiles in patients treated with risdiplam (patients aged 12–60 years)
  - To evaluate the efficacy of treatment with risdiplam in terms of patient-reported independence (patients aged 12–60 years) and caregiver-reported independence as assessed through the SMA Independence Scale–Upper Limb Module (SMAIS–ULM) total score in patients aged 2–60 years
  - To assess time to death (patients aged 1 year to < 2 years)
  - To assess time to permanent ventilation (patients aged 1 year to < 2 years).

**Supplementary results**

**Exploratory motor function endpoints**

- Exploratory motor function endpoints are reported separately for patients who had previously received nusinersen or onasemnogene abeparvovec.

**Additional exploratory endpoints**

**Patient-/caregiver-reported outcomes**

The caregiver-reported SMAIS–ULM was recorded for patients aged 2–60 years, and the patient-reported SMAIS–ULM also recorded for patients aged 12–60 years. SMAIS–ULM was added as an exploratory endpoint after the start of the study and as a result not all patients were assessed at baseline.

- A mean increase in caregiver-reported SMAIS–ULM total score was observed from baseline to month 24.
  - At month 24, the mean change from baseline in caregiver-reported SMAIS–ULM total score was 1.90 (95% confidence interval [CI] 0.70–3.10, *n* = 110).
- A stabilization in the patient-reported SMAIS–ULM mean total score was observed from baseline to month 24.
  - At month 24, the mean change from baseline in patient-reported SMAIS–ULM total score was –0.67 (95% CI –1.58 to 0.23, *n* = 83).

**Respiratory function**

As per the protocol, spirometry assessments were performed in patients aged 6–60 years old and the SNIP test was performed in patients aged 2–60 years.

Limited data for respiratory outcomes in patients with late-onset SMA are available. Assessments of pulmonary function are sensitive to various factors, including scoliosis, use of airway clearance, and respiratory tract infections [1, 2]. JEWELFISH enrolled a broad population of patients with high levels of scoliosis (83%) and severe scoliosis (39%). Pulmonary care use was reported by 52% of patients at baseline. All these factors may have affected the respiratory outcomes in the overall population. The most relevant exploratory respiratory endpoints for this population are reported.

**Spirometry**

- At month 24, the mean change from baseline in FVC (best percentage predicted) was –8.25% (95% CI –10.85 to –5.65, *n* = 100).
- At month 24, the mean change from baseline in PCF (best percentage predicted) was –0.59% (95% CI –2.33 to 1.14, *n* = 96).

**SNIP**

- At month 24, the mean change from baseline in SNIP (best percentage predicted) was 0.14% (95% CI –2.64 to 2.92, *n* = 113).

**Table S1** Exploratory efficacy motor function endpoints by previous treatment group

| **Motor function assessments in patients aged 2–60 years** | | | | |
| --- | --- | --- | --- | --- |
|  | | **Nusinersen (*n* = 76)** | **Onasemnogene abeparvovec**  **(*n* = 14)** | **All patients**  **(*N* = 174)** |
| **MFM32 total score**  **Month 12**  Mean change from baseline  (95% CI)  *n* | | –1.31  (–2.34 to –0.28)  59 | 4.82  (–1.21 to 10.85)  8 | –0.35  (–1.05 to 0.35)  138 |
| **MFM32 total score**  **Month 24**  Mean change from baseline  (95% CI)  *n* | | –1.25  (–2.55 to 0.04)  59 | 6.25  (0.31 to 12.19)  9 | –0.17  (–1.03 to 0.68)  137 |
| **RULM total score**  **Month 12**  Mean change from baseline  (95% CI)  *n* | | 0.32  (–0.40 to 1.04)  63 | 2.78  (–0.70 to 6.25)  9 | 0.16  (–0.32 to 0.63)  146 |
| **RULM total score**  **Month 24**  Mean change from baseline  (95% CI)  *n* | | 0.50  (–0.20 to 1.20)  58 | 6.11  (2.11–10.11)  9 | 0.71  (0.17–1.26)  133 |
| **HFMSE total score**  **Month 12**  Mean change from baseline  (95% CI)  *n* | | –0.27  (–1.15 to 0.61)  59 | 4.67  (1.41–7.93)  9 | 0.38  (–0.13 to 0.89)  140 |
| **HFMSE total score**  **Month 24**  Mean change from baseline  (95% CI)  *n* | | –1.21  (–2.79 to 0.36)  56 | 7.11  (3.93–10.29)  9 | –0.11  (–0.98 to 0.76)  132 |
| **Motor function in ambulant patients aged 6–60 years** | | | | |
|  |  | **Nusinersen**  **(*n* = 12)** | **MOONFISH**  **(*n* = 3)** | **Patients aged 6–60 years**  **(*n* = 12)** |
| **6MWT total distance walked^a^**  **Month 12**  Mean change from baseline  (95% CI)  *n* | | 21.56  (–13.96 to 57.07)  9 | - | 21.56  (–13.96 to 57.07)  9 |
| **6MWT total distance walked^a^**  **Month 24**  Mean change from baseline  (95% CI)  *n* | | 8  30.88  (–5.54 to 67.29)  8 | - | 8  30.88  (–5.54 to 67.29)  8 |

*6MWT* 6-min walk test, *CI* confidence interval, *HFMSE* Hammersmith Functional Motor Scale–Expanded, *MFM32* 32-item Motor Function Measure, *RULM* Revised Upper Limb Module

**^a^** The 6MWT was added as an exploratory endpoint for ambulant patients ≥ 6 years of age after the start of the study and therefore not all patients have baseline measurements

**Table S2** Post hoc analysis on exploratory efficacy motor function endpoints in patients aged 25–60 years

|  | **Month 12** | **Month 24** |
| --- | --- | --- |
| **MFM32 total score**  Mean change from baseline  (95% CI)  *n* | –0.89  (–2.46 to 0.68)  28 | –0.19  (–1.78 to 1.41)  28 |
| **RULM total score**  Mean change from baseline  (95% CI)  *n* | –0.45  (–1.75 to 0.85)  29 | 0.00  (–0.98 to 0.98)  27 |
| **HFMSE total score**  Mean change from baseline  (95% CI)  *n* | 0.34  (–0.50 to 1.19)  29 | –0.15  (–0.99 to 0.69)  27 |

Thirty-four patients in JEWELFISH were aged 25–60 years; 13 (38%) of whom were reported as Type 2 SMA and 21 (62%) Type 3 SMA. Twenty-two patients (65%) had an HFMSE total score of < 10 at baseline

*CI* confidence interval, *HFMSE* Hammersmith Functional Motor Scale–Expanded, *MFM32* 32-item Motor Function Measure, *RULM* Revised Upper Limb Module, *SMA* spinal muscular atrophy

**References**

1. Wijngaarde CA, Veldhoen ES, van Eijk RPA, Stam M, Otto LAM, Asselman FL, Wosten-van Asperen RM, Hulzebos EHJ, Verweij-van den Oudenrijn LP, Bartels B, Cuppen I, Wadman RI, van den Berg LH, van der Ent CK, van der Pol WL (2020) Natural history of lung function in spinal muscular atrophy. Orphanet J Rare Dis 15:88. <https://doi.org/10.1186/s13023-020-01367-y>.

2. Trucco F, Ridout D, Scoto M, Coratti G, Main ML, Muni Lofra R, Mayhew AG, Montes J, Pane M, Sansone V, Albamonte E, D'Amico A, Bertini E, Messina S, Bruno C, Parasuraman D, Childs AM, Gowda V, Willis T, Ong M, Marini-Bettolo C, De Vivo DC, Darras BT, Day J, Kichula EA, Mayer OH, Navas Nazario AA, Finkel RS, Mercuri E, Muntoni F, International SMA Consortium (2021) Respiratory Trajectories in Type 2 and 3 Spinal Muscular Atrophy in the iSMAC Cohort Study. Neurology 96:e587-e599. <https://doi.org/10.1212/WNL.0000000000011051>.
